# A metatranscriptomic analysis of diseased social wasps (*Vespula vulgaris*) for pathogens, with an experimental infection of larvae and nests

**DOI:** 10.1371/journal.pone.0209589

**Published:** 2018-12-31

**Authors:** Oliver Quinn, Monica A. M. Gruber, Robert L. Brown, James W. Baty, Mariana Bulgarella, Philip J. Lester

**Affiliations:** 1 Centre for Biodiversity and Restoration Ecology, School of Biological Sciences, Victoria University of Wellington, Wellington, New Zealand; 2 Pacific Biosecurity, Victoria Link Limited, Victoria University of Wellington, Wellington, New Zealand; 3 Biodiversity and Conservation, Manaaki Whenua–Landcare Research, Lincoln, New Zealand; University of North Carolina at Greensboro, UNITED STATES

## Abstract

Social wasps are a major pest in many countries around the world. Pathogens may influence wasp populations and could provide an option for population management via biological control. We investigated the pathology of nests of apparently healthy common wasps, *Vespula vulgaris*, with nests apparently suffering disease. First, next-generation sequencing and metatranscriptomic analysis were used to examine pathogen presence. The transcriptome of healthy and diseased *V*. *vulgaris* showed 27 known microbial phylotypes. Four of these were observed in diseased larvae alone (*Aspergillus fumigatus*, *Moellerella wisconsensis*, *Moku virus*, and the microsporidian *Vavraia culicis*). *Kashmir Bee Virus* (KBV) was found to be present in both healthy and diseased larvae. *Moellerella wisconsensis* is a human pathogen that was potentially misidentified in our wasps by the MEGAN analysis: it is more likely to be the related bacteria *Hafnia alvei* that is known to infect social insects. The closest identification to the putative pathogen identified as *Vavraia culicis* was likely to be another microsporidian *Nosema vulgaris*. PCR and subsequent Sanger sequencing using published or our own designed primers, confirmed the identity of *Moellerella* sp. (which may be *Hafnia alvei*), *Aspergillus* sp., KBV, *Moku virus* and *Nosema*. Secondly, we used an infection study by homogenising diseased wasp larvae and feeding them to entire nests of larvae in the laboratory. Three nests transinfected with diseased larvae all died within 19 days. No pathogen that we monitored, however, had a significantly higher prevalence in diseased than in healthy larvae. RT-qPCR analysis indicated that pathogen infections were significantly correlated, such as between KBV and *Aspergillus* sp. Social wasps clearly suffer from an array of pathogens, which may lead to the collapse of nests and larval death.

## Introduction

Social wasps can be a major pest in their introduced range. In countries such as New Zealand, *Vespula germanica* and *V*. *vulgaris* are considered to be amongst the most damaging and widespread invertebrate pests [1]. First observed in 1921, *V*. *vulgaris* has become especially abundant, most notably in New Zealand beech forests (*Fuscospora* spp.), with densities exceeding 370 wasps m^-2^ of tree trunk and up to 40 nests ha^-1^ [2,3]. They are major predators of native invertebrates, competitively excluding native birds and estimated to cost New Zealand’s economy more than NZ$133 million per annum. The economic costs particularly impact farming, beekeeping, horticulture and forestry workers, resulting in a focused effort to develop control strategies [4].

Vespid wasp colonies have previously been reported to suffer from pathogens [5]. Considerable research has been undertaken to find biocontrol candidates that could effectively be used to reduce populations of *V*. *vulgaris* in their new ranges [6–8]. From this research, many pathogens and parasites have been identified, however, to date no effective biocontrol has been successful and vespid wasps continue to proliferate in newly acquired ranges such as New Zealand and Argentina [4]. Recently, diseased wasps were found in a study investigating the association of the mite *Pneumolaelaps niutirani* with *V*. *vulgaris* in New Zealand [9,10]. Larvae in the infected nests showed signs of deterioration, presenting abnormal opaque discoloration beneath the cuticle indicating hypertrophied tissues. It was noted at the time that this apparent but undetermined pathogen appeared particularly virulent, causing infections to spread to other close-by experimental wasp nests housed in a glasshouse [10].

The increased potential of microbial control of insect pests over the past few decades has largely been the result of the discovery and development of new species and strains of entomopathogens [11]. These entomopathogens cause disease through the effects of infection, parasitism and/or toxaemia and may not always be apparent in the host through gross pathology. Koch's postulates have historically been used to establish a causative relationship between a pathogen and disease [12], and have been reconsidered and revised for sequence-based identification of microbial pathogens [13]. These revisions include that the putative pathogen should be found present in the majority of cases of an infectious disease, that the sequence copy number correlates with severity of disease or pathology, and that the sequence-based evidence for microbial causation should be reproducible [13].

The goal of this study was to identify potential candidate pathogens that may have caused pathology in common wasp (*V*. *vulgaris*) nests, and experimentally induce infection in a time series study in *V*. *vulgaris* nests and larvae. Larvae from mite-infested and disease-symptomatic nests were sampled in order to detect the pathogen causing the demise of the nests, with the hope of identifying a potential natural biocontrol for further study via molecular methods. We used Illumina Next Generation Sequencing (NGS) metatranscriptome analysis in addition to confirmatory Polymerase Chain Reaction (PCR) and Sanger sequencing methods. We also attempted to test the infectivity and virulence of pathogen candidates by orally infecting apparent healthy larvae and entire *V*. *vulgaris* nest colonies in the lab in a time series infection study.

## Material and methods

### Sample collection, RNA extractions, and metatranscriptomic comparison

Live diseased and healthy *V*. *vulgaris* larvae were sampled from three healthy and three diseased nests in April 2016 from the Lincoln area in Canterbury, New Zealand. No specific permission was required to collect the nests and common wasps in New Zealand are not considered an endangered or protected species. The diseased larvae were typical of those that have been frequently observed in New Zealand to present disease symptoms of unknown origin, with opaque tissues beneath the cuticle [10]. Larvae in the early stages of infection frequently had obvious white masses (<1mm in diameter) under their cuticle. Larvae in later stages of infection turned grey and exhibited reduced movement, before dying. The adult wasps in nests with infected larvae were substantially less aggressive in their defence of the colony, to the extent that by using aggression levels of nests observed in the field it was possible to predict which nests were infected and carried diseased larvae prior to excavation. The healthy larvae and apparently uninfected nests lacked these white masses, with adults actively and aggressively defending their colonies. The larvae from each nest were snap frozen at -80°C for subsequent RNA extraction and analysis.

RNA-Seq metatranscriptomic analysis was undertaken to investigate candidates for the cause of the infection, through a comparison of healthy and diseased larvae. Total RNA was extracted from three larvae from three nests presenting disease symptoms, and single larvae from three nearby nests that appeared healthy and without disease symptoms. RNA was extracted by bead-beating (BeadBeater 16, Biospec Products, USA) samples in GENEzol reagent (Geneaid, Taiwan) with 5% *β*-mercaptoethanol, followed by chloroform and isopropanol purification. RNA integrity was confirmed and quantified with an RNA 6000 Nano chip on an Agilent 2100 Bioanalyser (Agilent Technologies Co. Ltd., Diegem, Belgium) according to the manufacturer’s instructions. RNA samples were then sent for Illumina RNA-seq. Samples, were sequenced as 125 bp paired end barcoded Trueseq libraries (Macrogen, South Korea), using Illumina Hiseq. Post-processing quality control excluded bases with quality scores Q > 30, and trimmed adapters. Illumina RNA-seq data were assembled using Trinity v 2.0.6 with read normalisation [14]. Assemblies were run on the Victoria University of Wellington Science Faculty's High Performance Computing Facility.

To select potential novel pathogen sequences in the assemblies, metatranscriptomic contigs from both diseased and healthy larvae were analysed. We first aligned sequences (e-value of 0.001) to a binary non-redundant (nr) protein database [15] using DIAMOND for faster processing times [16]. Taxonomic identity was annotated in MEGAN6 community edition (Version 6.8.18) [17]. The meganize function in MEGAN6 assigned taxonomy based on 97% identity to accession files. The resulting DIAMOND files were visualised and compared in MEGAN6 [17]. BLASTx protein alignments were used to identify putative viral, microbial eukaryote and bacterial proteins. These aligned candidate sequences were confirmed by extracting sequences from the assembly file and searched using blastx of the Genbank NCBI nr database.

Viral particles were imaged using a Transmission Electron Microscope (TEM). Diseased larvae were dissected, dehydrated and fixed (stabilized) in hard resin. Using an ultramicrotome ultra-thin slices (100 nm or thinner allowing electrons to pass through the sample) were cut from the tissue embedded in resin and placed on a TEM grid. Samples were then treated with direct negative stain heavy metals (uranyl acetate) to increase the level of contrast in the final image. Samples were then imaged on a Jeol JEM 2011 operating at 200 kV.

### Experimental infection of wasp nests and larvae

For the infection study, six subterranean *V*. *vulgaris* nests were extracted from several closely spaced sites within a 5 km radius in Wainuiomata, New Zealand, during April 2016. Each live nest was transferred to buckets in the field and cooled in a -20°C freezer for one hour to immobilize but not kill stinging workers.

Two combs containing fourth and fifth instar *V*. *vulgaris* larvae were removed from each nest for the larval infection experiment. One wasp comb was designated control while the other was used for treatment. All combs were placed in storage containers with ventilation in a separate incubator kept at 27°C, 24 hr darkness and ~60% RH. Combs were glued to the lid of each storage container, so that when placed back on the box, combs faced downwards, mimicking the orientation in the natural environment (S1 Appendix).

The remaining combs and wasp were then transferred and sealed into a predesigned and fabricated wooden box (40 × 40 × 40 cm). Three nests each were randomly designated for pathogen infection and three used as control nests. Each nest was placed on a wire mesh hammock for suspension, to allow nest repair and continued growth. A foraging box arena was attached to the nest box by a 1.5 m transparent pipe, where wasps were allowed to leave the nest box to forage in an additional plastic foraging box (25 × 30 × 40 cm). The foraging arena was where food was located and enabled monitoring of the individual wasps for the duration of the experiment (S1 Appendix). All nests were acclimated for 2 days in a 27°C environmentally controlled room, with conditions of 16:8 light:dark cycle, and ~60% RH, where they were fed a 50:50 water and honey solution daily in the foraging arena *ad libitum* through a mesh opening drip fed onto a petri dish on foraging arena floor. Additional replicate nests would have been ideal in this experimental design, though we were limited in our experimental design by space and nest availability constraints.

We then sought to experimentally infect the wasp nests with disease 3 days after collection. Wasp larvae from the nests used in the transcriptomic work, showing the typical signs of disease as described above were used in this analysis. Apparent healthy larvae were also collected. Three diseased (~2.5g) and three healthy larvae (2.5g) were homogenised separately under sterile conditions using Qiagen TissueRupter (Hilden, Germany) in a laminar flow hood. Homogenisation of healthy larvae, or diseased larvae, was carried out in a 50:50 honey (6 mL) and water (6 mL) solution (v/v). The treatment (infected) diet was then administered to the treatment wasp nests and individual test larvae. The control (healthy) diet was administered to the control wasp nests and individual control larvae. Homogenates were not tested for pathogens.

Each diet was fed directly to whole nests by placing the solution into the foraging arena through the mesh opening and drip fed onto a petri dish on the foraging arena floor. A subset of each diet was individually administered orally (5 μL, by pipette) to each larva in both test and control combs by pipette. These larvae were a mixture of 3^rd^ to 5^th^ instar wasps. This measurement of 5 μL was based on larvae reportedly regurgitating up to 5 μL of saliva in a single trophallactic exchange [18]. For the remainder of the experiment all nests and larvae were fed daily 50:50 water and honey with homogenised healthy larvae as a protein source. Nests were examined daily for wasp larvae movement and survival. Sampling of three larvae from control and treatment combs was carried out for the duration of the experiment (12 days). We used R version 3.3.1 [19] for all statistical analyses described below. The packages survival [20] and ggfortify [21] were used to examine for differences in survival between nest treatments. We acknowledge the small number of colonies in this analysis, though given the novelty of the approach we believe it to be indicative and useful for future work.

Treatment and control larval samples were analysed by reverse transcriptase PCR (RT-PCR to confirm candidate targets, and by RT-quantitative PCR (RT-qPCR) for time series analysis of pathogen expression in conjunction with one immune gene, *Dicer*. Total RNA was extracted from larvae sampled from treatment and control combs at each time point (1, 4, 5, 6, 7, 8, 10 and 12 days post-infection). A NanoDrop spectrophotometer (Thermo Fisher Scientific Inc., Waltham, USA) was used to measure RNA concentrations and 1 μg/sample was used for cDNA synthesis (SuperScript IV, Invitrogen/Thermo Fisher Scientific, Waltham, USA).

PCR primers were designed using Primer-BLAST [22] and used to confirm candidate targets. In addition, *Dicer* expression was used to test immune gene expression in relation to viral phylotype load. *Dicer* was chosen based on its role in RNA viral immunity. The expression of this immune gene has previously been found to be associated with viral infection and wasp nest fitness [23]. PCR reactions were prepared with 2 μL (100 ng) cDNA/reaction, primers and REDTaq DNA polymerase (Sigma Aldrich). Cycling conditions were: 95°C, 2 mins; 40 cycles of 95°C, 20s; 60°C, 20s; 72°C, 30s; followed by 72°C, 5 mins. Where PCR produced clear bands, products were treated with ExoSAP-IT (Affymetrix USB products, USA) and sequenced (Massey Genome Service, Palmerston North, New Zealand). Sequences were checked and aligned using MEGA6 [24].

For the time series RT-qPCR analysis, each sample was analysed in duplicate using 1 μL (50 ng) cDNA/reaction, primers and PowerUp SYBR Green Master Mix (Applied Biosystems/Thermo Fisher Scientific, USA. All qPCR reactions were run on QuantStudio 7 Flex Real-Time PCR System (Applied Biosystems/Thermo Fisher Scientific, USA) using fast cycling conditions (95°C, 30s; 40 cycles of 95°C, 5s; 60°C, 15s; 72°C, 20s). Cycle threshold (C*t*) values were used to calculate target levels relative to the reference genes (eIF3-S8 and Pros54; [23]) using the equation (2^(-C*t*_target_))/(2^(-C*t*_reference_)). To account for variation in sample quality, an upper Cq threshold of 35 was set, above which values were excluded from quantitative analysis. The use of C*t* values in this fashion is a standard approach for viral quantification in hymenopteran insects and in other systems [25]. While we note that in some work reference gene expression appears unstable [26], we found no evidence that the two reference genes in our study were not stably expressed.

To assess differences in pathogen prevalence and *Dicer* expression in larvae from the control and treatment groups after inoculation, we used a one-way ANCOVA in R [19]. We included time since infection as a covariate. The response variables were log-transformed. We used the package PerformanceAnalytics [27] to produce correlation statistics and plots to examine correlations between pathogens and the immune gene *Dicer* expression.

## Results

### Metatranscriptome study

**Fig 1:**
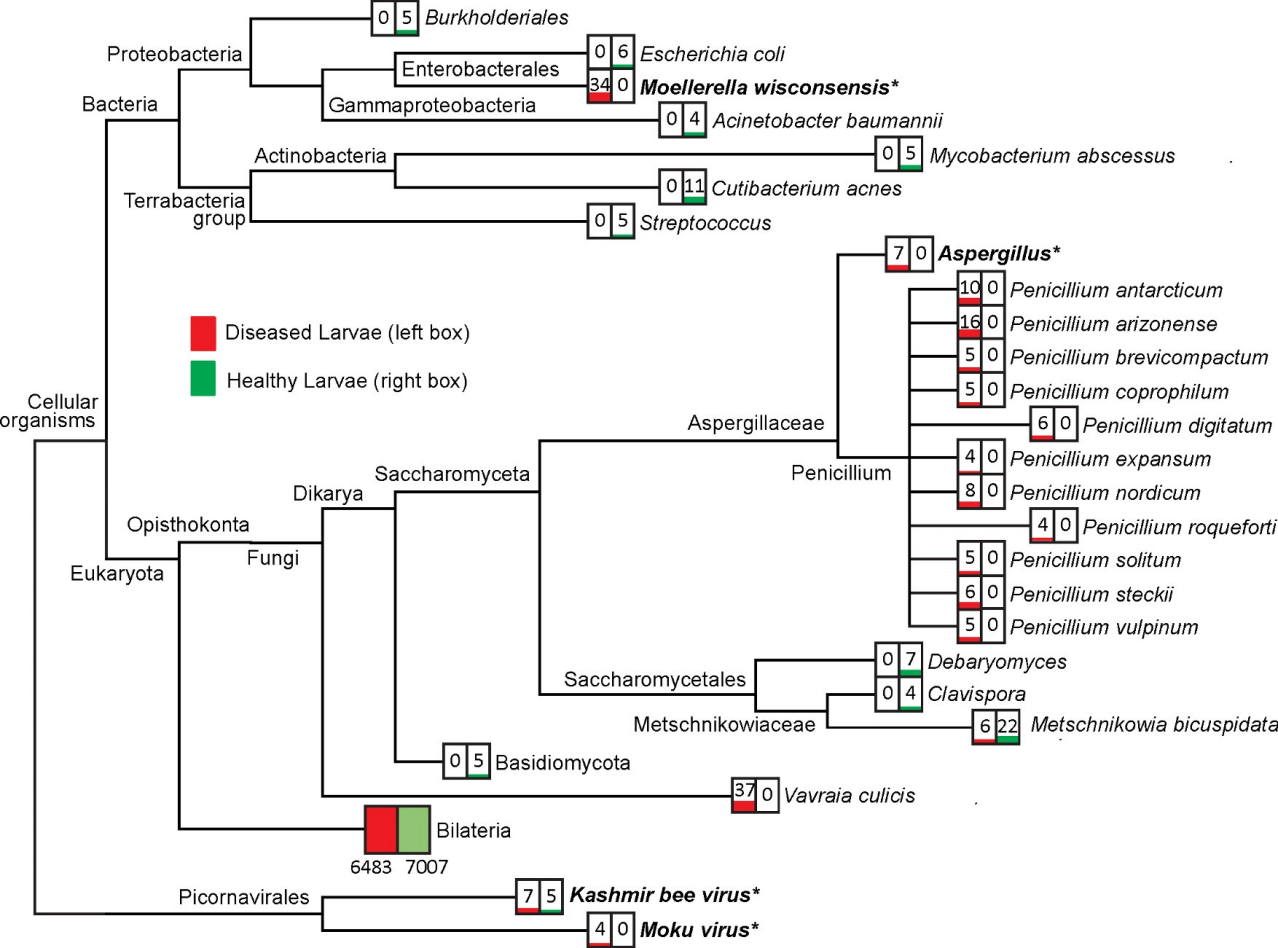
Phylogenetic tree comparison of diseased and healthy larvae microbiota constructed in MEGAN. This visual representation details the metatranscriptome sequence reads that aligned at 97% identity to known sequences in NCBI nt and nr databases. These alignments to taxa represented here are the most likely candidates. Lowest taxa identified are displayed with the number of read sequences aligned healthy larvae (green) and diseased (red). Bilateria sequences include all host sequences, the majority of which aligned to sequences from the wasp *Polistes dominula* and *P*. *canadensis* genomes. These genomes are the most closely related annotated wasp genomes to *V*. *vulgaris*.

We performed metatranscriptomic comparative analyses using RNA-seq to elucidate the cause of *V*. *vulgaris* larvae symptomatic of an unknown infection. An average of approximately 116,000 paired end sequences (contigs) from each fasta file aligned and were annotated using Diamond against the NCBI database. The *meganize* function in MEGAN6 assigned taxonomy based on 97% identity to accession files provided. The improved LCA algorithm used in Diamond deals with a "minimum taxon cover" which greatly enhances the specificity of the taxonomic LCA placement algorithm. The specificity and taxonomic certainty of the metatranscriptomic comparative analysis identified matches to 27 known microbiota phylotypes in both healthy and diseased communities (Fig 1). Differences in the comparison analysis highlighted five potential pathogen candidates, the bacterium *Moellerella wisconsensis*, the fungus *Aspergillus fumigatus*, the microsporidian *Vavraia culicis* and two RNA dicistroviruses, *Kashmir Bee Virus* (KBV) and *Moku virus*. The first four of these candidates were identified in the diseased larvae alone, with KBV identified in both diseased and healthy wasps. Sequences were extracted and primers were designed to further investigate and confirm the identity of these five candidates. The metatranscriptome analysis also highlighted the genus *Penicillium* found only in diseased larvae, of which eleven species were identified. In general, *Penicillium* fungi are considered non-pathogenic [28] therefore this genus was not investigated further. Taxa identified as present in healthy larvae and absent from diseased larvae consisted of *Burkholderiales* spp., *Escherichia coli*, *Acinetobacter baumannii*, *Mycobacterium abscessus*, *Cutibacterium acnes*, *Streptococcus* spp., *Debaryomyces* spp., *Basidiomycota* spp. and *Metschnikowia bicuspidate*.

**Fig 2:**
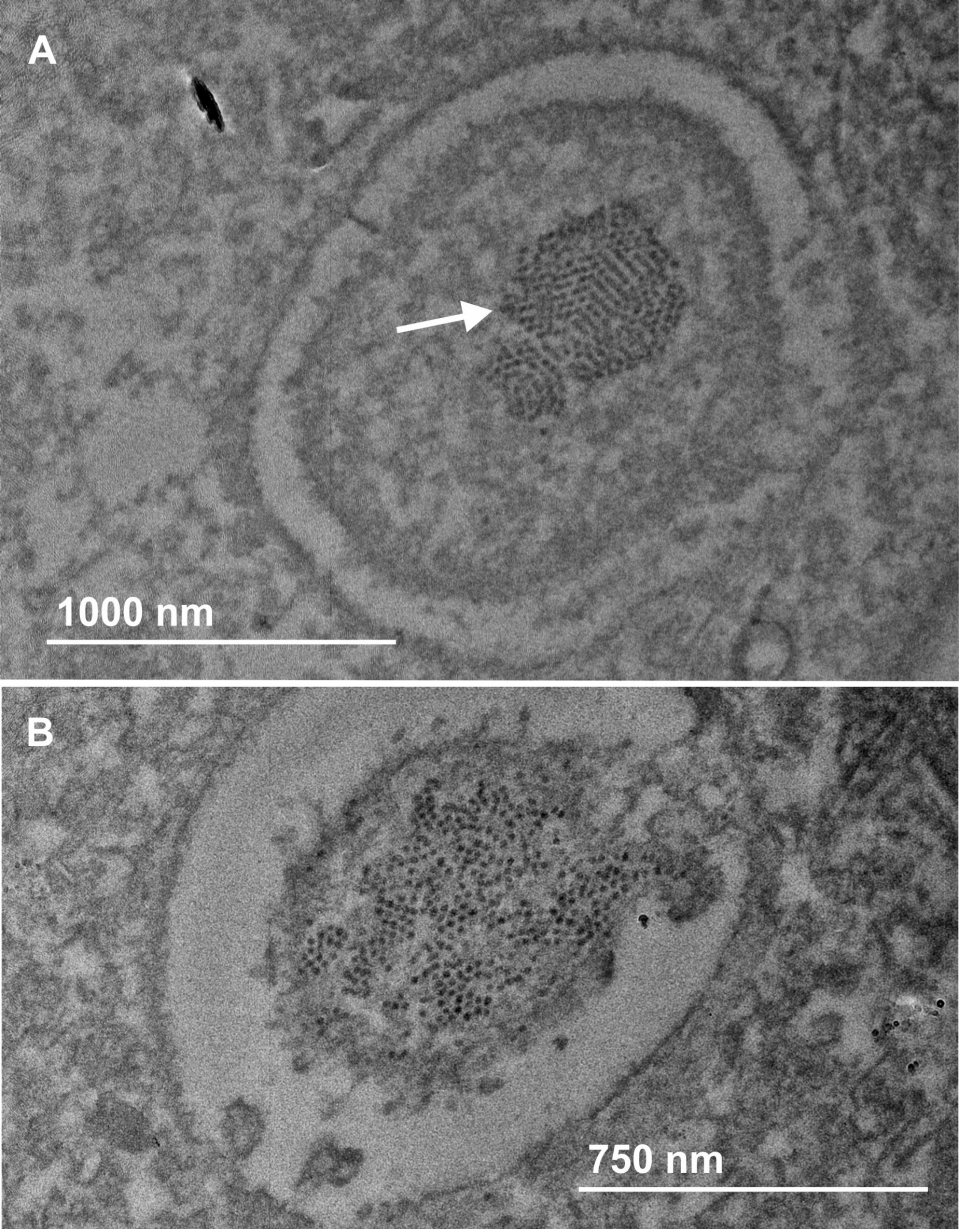
(A) Electron microscope image of virus particles forming a crystalline array within the nucleus of a wasp cell. These virus particles are likely to be KBV, given the prevalence of this virus in the wasps and their icosahedral shape typical of this dicistrovirus. (B) Electron microscope image showing cell lysis and release of viral particles into the surrounding wasp tissue further infecting and replicating in healthy cells. Photos by David Flynn (School of Chemical and Physical Science, Victoria University of Wellington, Wellington, New Zealand).

To confirm the potential pathogen identity and analyse pathogen load, RT-qPCR primers were designed for *M*. *wisconsensis*, *A*. *flavus*, *V*. *culicis*, KBV and *Moku virus*. PCR products were sequenced and their identity confirmed using MEGA to align to similar sequences on Genbank at 97% identity. Viral candidates *Moku virus* and KBV were further confirmed using long-range primers, resulting in longer products for sequencing (744 and 690 bp, respectively) and alignment. The TEM analysis appeared to indicate a viral infection within the nuclei of wasp cells (Fig 2). Analysis of nucleotide sequences confirmed the identity of all candidates apart from *V*. *culicis*, which was based on alignment to a *V*. *culicis* hypothetical mRNA protein (XM_008075167.1). Therefore, despite its apparently high abundance in the diseased wasp samples, our attempts to refine an analysis of this target were unsuccessful and it was not investigated further. The candidate sequence *M*. *wisconsensis* also aligned to *Hafnia* sp. (98% identity) and *Hafnia alvei* (97% identity), which is a related bacterium.

Given the limited knowledge of bacteria and pathogens in these wasps, we are not confident that the bacterium identified is actually *Moellerella wisconsensis* or that the fungus is *Aspergillus fumigatus*. Typically, *A*. *fumigatus* is described as a pathogen of mammals that does not cause mortality to insects after injection [29]. Similarly, *M*. *wisconsensis* is a known human pathogen [30]. Consequently from this point forward we refer to each as *Moellerella* sp. and *Aspergillus* sp., respectively.

### Experimental infection of wasp nests

**Fig 3:**
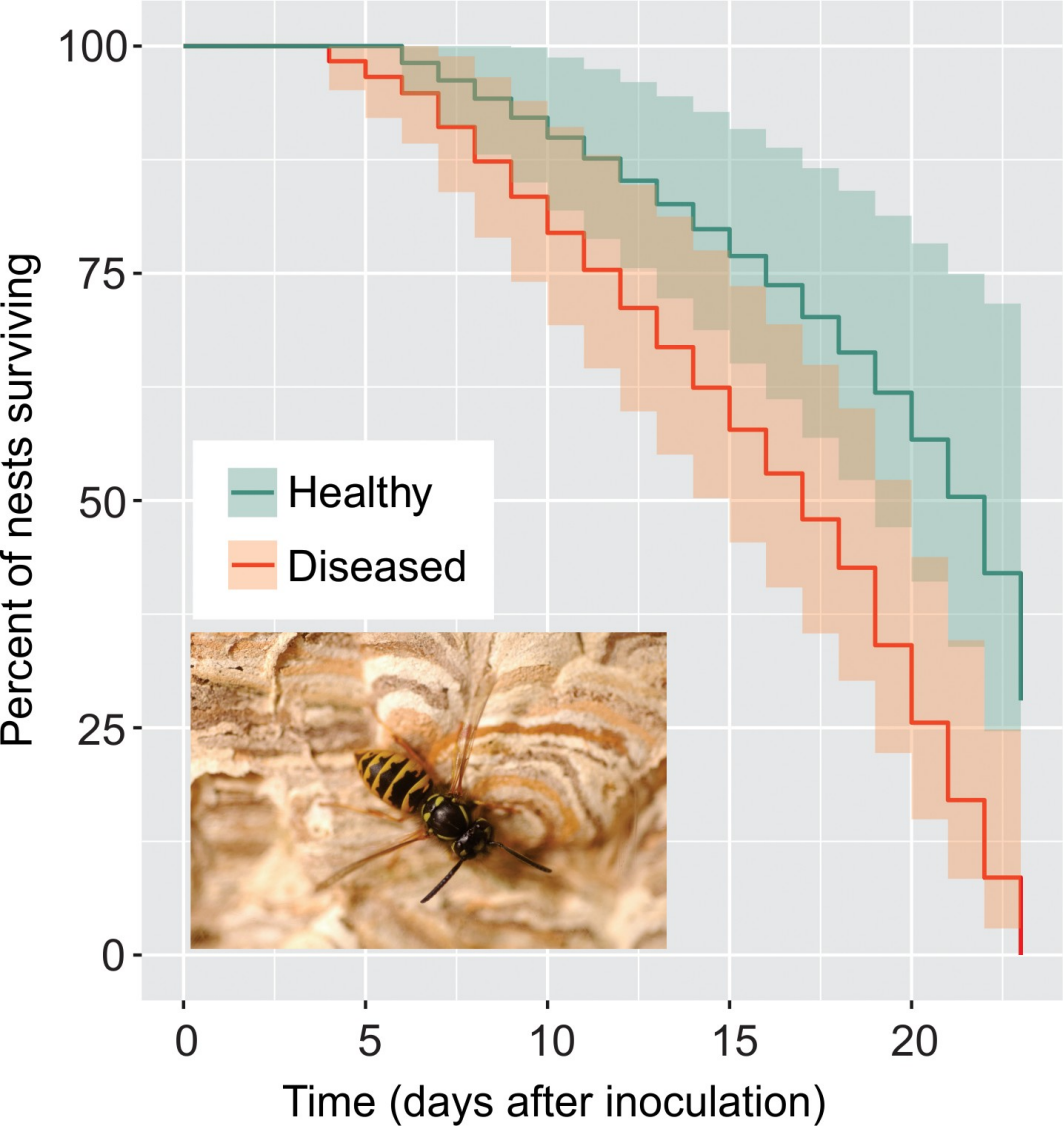
Kaplan–Meier survival curves showing the percentage of nest survival over the duration of the study (days). Green indicating control nests that were inoculated with homogenized wasp larvae from a separate and apparently healthy nest, and red indicating nests that feed homogenized larvae from a separate wasp nest showing signs of disease. Shaded areas represent the 95% confidence intervals, n = 3 nests per treatment. A Cox's proportional hazards model indicated a significant difference between the treatments (ß = -0.913 ± 0.283 SE; exp(ß) = 0.401; *P* = 0.001). Inset photograph is of an adult worker common wasp on her nest (photo by Phil Lester).

Whole wasp nests were fed either homogenised wasp larvae presenting infection symptoms (treatment), or the same weight of homogenized larvae from an apparently healthy nest (control). Two of the three control nests survived the entire duration of the study, while the third untreated nest died 6 days after treatment. This nest was relatively small and nest dissection at the end of the experiment indicated it produced only males, which do not forage or feed themselves, indicating that the queen had likely died prior to the nest collection in the field. Without a queen colony death would be expected if only males are produced because they do not forage or contribute to the colony maintenance. All the nests in the infection treatment appeared large and healthy at the beginning of the study. These treated nests infected with diseased larvae died after 4, 7 and 19 days. A Cox Proportional Hazards model indicated a significant difference in the survivorship of the control and treated nests (*ß* = -0.913 ± 0.283 SE; exp(ß) = 0.401; *P* = 0.001) (Fig 3).

We were, unfortunately, unable to monitor for disease within the nests over the duration of the experiment because such sampling would require substantial disturbance in the form of anesthetising the entire colony, and then breaking it apart to sample larvae.

### Experimental infection of wasp larvae

**Fig 4:**
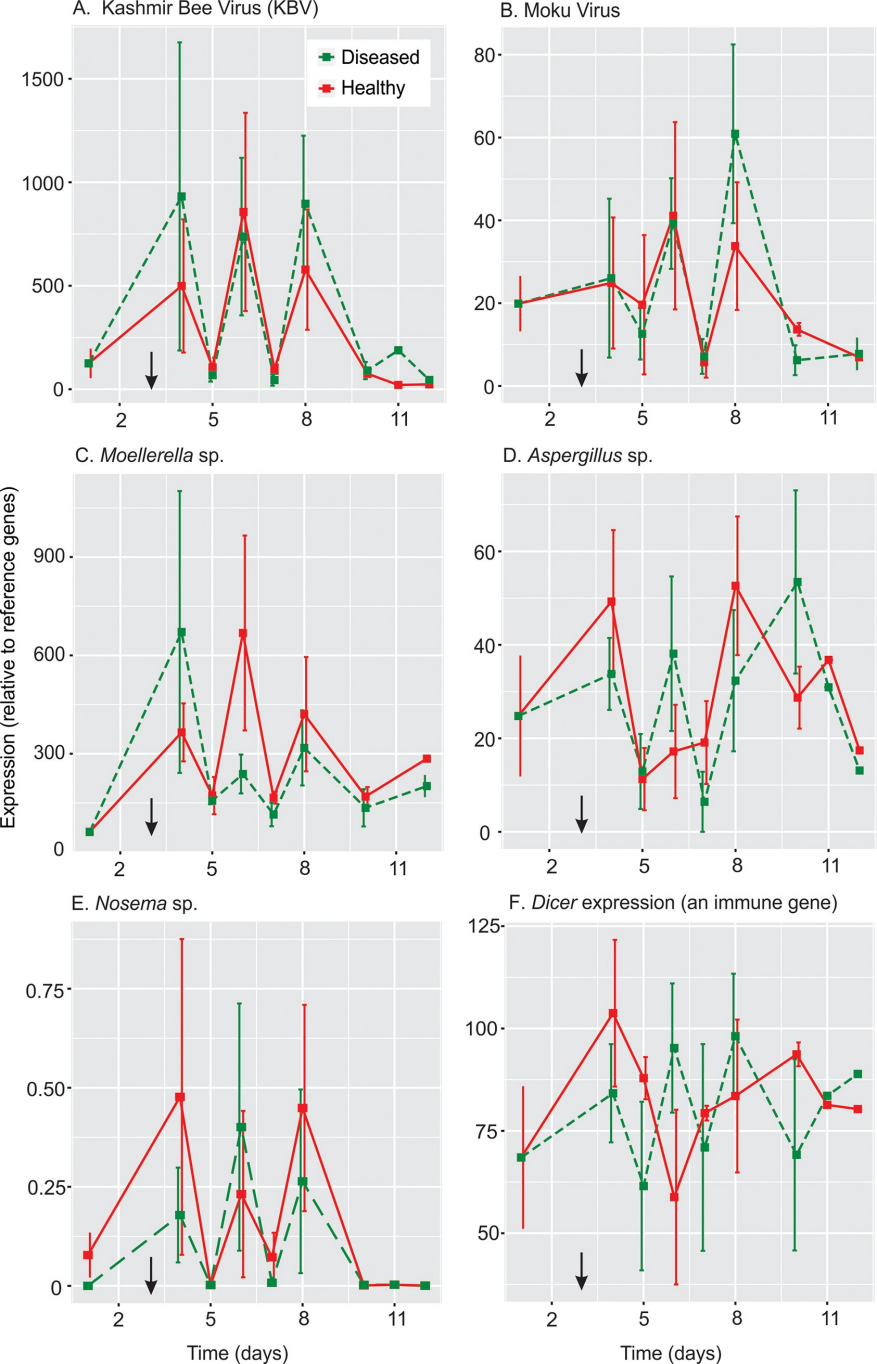
**The mean prevalence expression of (A) *Kashmir Bee Virus*, (B) *Moku virus*, (C) *Moellerella* sp., (D) *Aspergillus* sp., (E) *Nosema* sp., and (F) the immune gene *Dicer* in *V*. *vulgaris* larvae, relative to the reference genes *Pros54* and *eIF3-S8*.** Mean relative expression was achieved averaging the target RT-qPCR cq values from larvae sampled from four separate nest combs at each time point. The inoculation of homogenised diseased larvae, or homogenised healthy larvae for the control, on day 3 is signified by the black arrow. Three larvae were sampled at each time point from control and test combs extracted from four *V*. *vulgaris* nests.

Following the infection study of treated and control larvae and subsequent RT-qPCR of sampled larvae, we quantified pathogen prevalence and *Dicer* immune expression over time. The one-way ANCOVA indicated that that prevalence of the four target pathogens and *Dicer* expression did not significantly increase following time of infection (Fig 4; Table 1). Surprisingly, all larvae showed expression of all bacteria and viruses that we examined, though with considerable variability over time. The expression of KBV, for example, seemed to fluctuate substantially from low levels of infection on day 5, to much higher levels on day 6, then back again to a low infection on day 7 (Fig 4A). *Moku virus*, *Moellerella* sp. and *Nosema* sp. showed a similarly fluctuating pattern (Fig 4). KBV typically was present in an order of magnitude higher levels than *Moku virus*.

**Table 1:** Results of one-way ANCOVA analyses comparing the relative infection of the five putative pathogens and *Dicer* expression over time, between treatment and control larvae. Interactions were all not significant (*P >* 0.05) and are not reported. Data were log(x+1) transformed prior to analysis.

Target		Estimate (± SE)	t Statistic	*P*-value
*Kashmir Bee Virus* (KBV)	Treatment	-0.270 (1.093)	-0.246			0.806
	Time	-0.051 (0.112)	-0.454			0.652
*Moku virus*	Treatment	-0.028 (0.096)	-0.294			0.770
	Time	0.203 (0.983	0.207			0.836
*Moellerella* sp.	Treatment	0.185 (0.637)	0.290			0.773
	Time	0.089 (0.065)	1.380			0.173
*Aspergillus* sp.	Treatment	-0.035 (0.954)	-0.037			0.971
	Time	0.084 (0.098)	0.865			0.390
*Nosema* sp.	Treatment	-0.013 (0.065)	-0.207			0.8366
	Time	-0.010 (0.012)	-0.880			0.383
*Dicer* expression	Treatment	0.013 (0.702)	0.019			0.985
* *	Time	0.023 (0.072)	0.322			0.748

**Fig 5:**
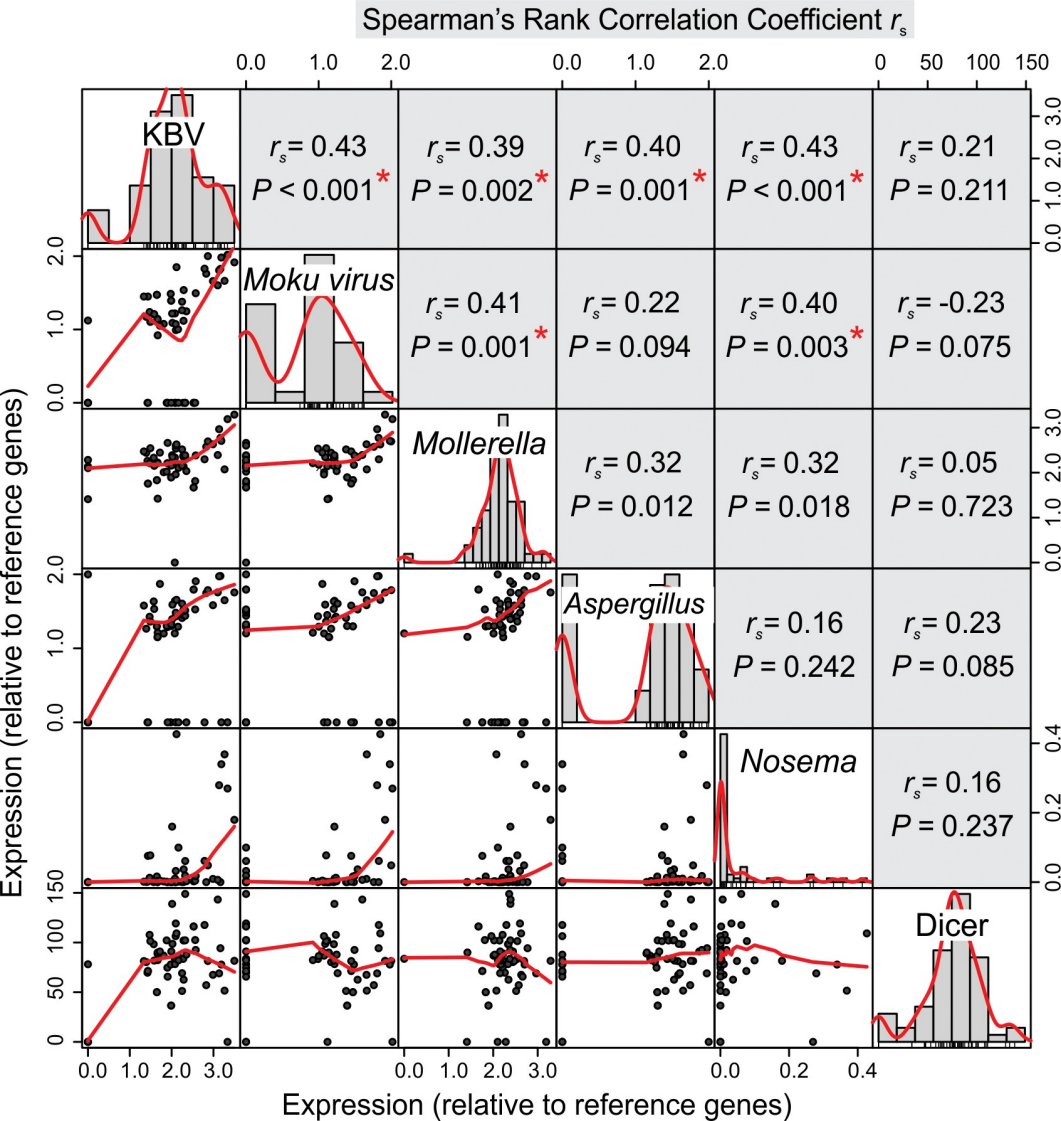
Correlations between the five potential pathogens and the immune gene *Dicer*, quantified using RT-qPCR in larval wasps. The frequency distribution of each is shown in the central diagonal. The scatterplot is shown with a loess fit in red and on the corresponding side for each pairing is the Spearman rank correlation coefficient *r*_s_. A Bonferroni correction was applied to the analyses and statistical significance is denoted by: *, which is *p* < 0.003 (or 0.05/15);. Values for all pathogens were log(x+1) transformed prior to analysis.

Because there were no significant differences in expression values between treatments, we combined the data to analyse for correlations between pathogens and the immune gene *Dicer* (Fig 5). Six of the 10 correlation analyses between pathogens showed significant positive correlations after a Bonferroni correction was applied (*p* < 0.003; or 0.05/15). KBV was correlated with all other pathogens examined (Fig 5). The Spearman rank correlation coefficient *r*_s_ between KBV and *Moku virus* was 0.43 (*n* = 59; *p <* 0.001), though when all 0 values were removed so that relationship was examined only when both viruses were present, this value increased to 0.61 (*n* = 41; *p <* 0.001). Similarly, the correlation coefficient between KBV and *Aspergillus* sp. increased from 0.40 (*n =* 59; *p <* 0.001) to 0.67 (*n =* 44; *p <* 0.001) when analysing data only when both species were detected. None of the pathogens were significantly correlated with patterns of *Dicer* expression.

## Discussion

The common wasp, *Vespula vulgaris*, has previously been reported to host a range of potential pathogens [8,23,31,32]. The experimental infection of entire wasp colonies in the laboratory resulted in the complete mortality of initially healthy nests after 4, 7 and 19 days. This experiment demonstrated that the pathogens responsible are infectious and can kill both adult and juvenile wasps. Few researchers have experimentally infected wasps with pathogens, though the published work that exists supports our observed mortality timelines. Two other studies infecting wasp workers with *Aspergillus* have found disease symptoms within 24 h, substantially reduced pupation rates, and adult mortality in as short a period as 2 days [7,18]. Other bacterial pathogens such as *Beauveria bassiana* and *Metarhizium anisopliae* can also kill workers in less than 10 days [18,33]. We would have liked to have additional experimental nests and to have been able to sample the larvae within the nests during the experiment. Nevertheless these experiments demonstrated that the pathogen involved is infectious, present in larvae, and is pathogenic across all wasp life stages and entire nests.

Our metatransciptomic approach identified four potential pathogen candidates that were associated with larvae sampled from diseased nests. These candidate pathogens were successfully sequenced and aligned to *Aspergillus* sp., *KBV*, *Moku virus*, *M*. *wisconsensis* and *Nosema* sp.. The candidate *M*. *wisconsensis* also aligned to *Hafnia* sp. (98% identity) and *Hafnia alvei* (97% identity), which are closely related to each other in addition to *Providencia* species (Hickman-Brenner et al., 1984). *Moellerella wisconsensis* known to be pathogenic in humans and other vertebrates including sharks [30,34] but is not known from invertebrates. However, *Hafnia alvei* has been found to occur in codling moth *Cydia pomonella*, *B*. *terrestris* and *A*. *mellifera*, where it has been highlighted as an opportunistic environmental bacteria [35–37]. Therefore, it is likely that the MEGAN analysis misidentified *M*. *wisconsensis* due to the close relation with *Hafnia* sp. *Moku virus* was recently discovered and described from wasps (*Vespula pensylvanica*), Honeybees (*Apis*. *mellifera*) and mites (*Varroa destructor*) on the Big Island of Hawai’i [38]. It is unknown what effects *Moku virus* has on their hosts. However, it was suggested that *Moku virus* has the potential to be highly virulent in *A*. *mellifera*, as is the closely related *Slow bee paralysis virus* [38]. This is the first known incidence of *Moku virus* in *V*. *vulgaris*, and also in New Zealand.

KBV was also identified in the MEGAN analysis as present in both healthy and infected larvae. KBV has been identified in a number of social insects and seems to be a commonly found pathogen in *V*. *vulgaris*, honey bees and even invasive ants in New Zealand [23,31,39,40]. KBV has also been described as part of a pathosphere implicated in honey bee colony collapse disorder [41] and can be extremely virulent when in high titres [42]. We found that KBV was present in wasp larvae at titres often an order of magnitude higher than *Moku virus*. However, it is still unclear as to the pathogenicity of KBV in these *V*. *vulgaris* samples, as it may potentially be latent virus [43].

The fungal pathogen *Aspergillus* sp., has previously been identified as a pathogen of vespid wasps [7] and are also part of the known pathosphere of honeybees [41]. *Aspergillus* are generalistic and opportunistic pathogens that often require wounds or weakened hosts [29]. *Aspergillus* are known to release mycotoxins and cause the bee larval disease stonebrood [41]. The disease symptoms are similar to those we observed in infected wasp larvae. In New Zealand, the collapse and death of wasp nests has been associated with the mite *Pneumolaelaps niutirani* [9,10]. This genus of mites is a known detritivore. They have, however, also been observed feeding on secretions around the mouths of wasp larvae. *Aspergillus* are known symbionts of detritivorous mites that may contribute to mite micronutrient diet deficits [44]. It is possible that the mite transmits *Aspergillus* during this feeding, though any *Aspergillus* infection may still be secondary and opportunistic after wounding by other pathogen(s). It remains to be determined if *Pneumolaelaps* mites do transmit *Aspergillus*, KBV, or any other pathogen. The correlations we observed between pathogens, including KBV and *Aspergillus*, may support an opportunistic and highly correlated infection pattern by wasp pathogens.

The metatranscriptome analysis also highlighted the microsporidian *Vavraia culicis*, which we believe to be a species of *Nosema* (perhaps *Nosema vespula*). *Vavraia* infections can be induced by oral feeding and has a very broad host range that includes shrimp, beetles and butterflies [45]. *Nosema* has been previously found in wasps [32,46]. Attempts have been made at using *V*. *culicis* for biological control of mosquitos. Microsporidian pathogens including *Vavraia* can reduce adult survival, increase adult female mosquito age-dependent mortality, and reduce female fecundity [47]. MEGAN analysis identified this candidate only in diseased larvae, but the experimental nests indicated it was present in both control and treated nests. These differing results between the metatransciptomic and infection experiments was due to the use of different wasp nests and samples for each experiment. The infection experiment, however, suggests that this microsporidian pathogen was not responsible for nest mortality.

The isolation and experimental infection of larvae was designed to enable a close examination of pathogen dynamics over time. The experiment required the removal of combs, positioning them in containers, and the daily artificially feeding the larvae. No contact with adult wasp workers was allowed. The results showed no clear differences in pathogen abundance between larvae that were fed a homogenate of diseased or healthy larvae from other nests. The absence of workers may have resulted in unhygienic conditions for larvae. Post feeding, on a number of occasions, both control and test larvae were observed on the bottom of the storage container having abandoned their comb cells and died. This unnatural environment may have stressed larvae incurring increases in opportunistic pathogens as seen in this study. Typically worker wasps carry out domestic duties including the distribution of food to larvae, nest cleaning and clearing of comb cells [5]. Therefore, maintaining larvae in a lab situation may need to include workers to ensure unnatural surroundings are not the cause of stress and disease, and mimic a more natural response. It is also conceivable that infected larvae were suffering from the inoculum received in this study and may have been presenting disease symptoms from a pathogen not tested by us. Larvae dropping from their cells is a symptom of disease [5]. Overall, the results from this experimental infection were inconclusive. It is clear from the transcriptomic work that potential pathogens do occur in these wasps and the entire nest infection experiment provides evidence that entire colonies can quickly die after infection. However, we may have not have monitored the responsible pathogen in our time-series, or issues associated with the artificial rearing of larvae may not enable researchers to identify responsible pathogens using these methods.

It is clear from our study, that an array of pathogens are present in *V*. *vulgaris*. Primary infection on average can generate more than one secondary infection by weakening the host, allowing coinfections of pathogens capable of invading the hymenopteran hosts (Otterstatter & Thomson, 2006). These wasps have a ‘pathosphere’ [41] that includes pathogens found in many other organisms. Some may be symbiotic in nature while others are potential pathogens that can decimate a nest.

## Supporting information

S1 Appendix S1_Supporting methods and results. (DOCX)
