# Supplementary material for: A metatranscriptomic analysis of diseased social wasps (Vespula vulgaris) for pathogens, with an experimental infection of larvae and nests
Source: PLoS One. 2018 Dec 31;13(12):e0209589. doi: 10.1371/journal.pone.0209589 (PMC6312278; doi:10.1371/journal.pone.0209589)
Supplement: S1 Appendix — (DOCX) [file pone.0209589.s001.docx]

# Supplementary Material:

# A metatranscriptomic analysis of diseased social wasps (*Vespula vulgaris*) for pathogens, with an experimental infection of larvae and nests

Oliver Quinn ^1^, Monica A.M. Gruber ^1,2^, Robert L. Brown ^3^, James W. Baty ^1^, Mariana Bulgarella ^1^, Philip J. Lester ^1,2,^*

*^a^ Centre for Biodiversity and Restoration Ecology, School of Biological Sciences, Victoria University of Wellington, PO Box 600, Wellington, New Zealand*

*^b^* [*Biodiversity and Conservation*](https://www.landcareresearch.co.nz/about/people/science-teams/biodiversity-and-conservation/)*, Manaaki Whenua – Landcare Research, P.O. Box 69040, Lincoln 7640, New Zealand*

*Corresponding authors: phil.lester@vuw.ac.nz, oliver.quinn@vuw.ac.nz,

Figure S1. Figure displaying nest combs with scale bar. Food was administered daily using a 10µL pipette, feeding each larvae individually. Nest combs are glued to the lids of a storage container and then placed on storage container box in the downwards position, mimicking comb orientation in the nest.

Figure S2. Figure showing glued nest combs to the lid of a storage container. All combs were orientated downwards, so when placed back on the box, combs mimicked the natural orientation in the nest.

Figure S3. Wooden nest box connected to foraging arena. Once two combs were retrieved the remaining nests were transferred and sealed into a predesigned and fabricated wooden box three nests designated test and three control. Each nest was placed on a wire mesh hammock so as suspended to allow nest repair and continued growth. The wooden nest box was connected by a 1.5m transparent pipe to a foraging arena, where wasps travelled to back and forth, as seen in the picture.

Table S1 Table detailing oligonucleotide primers designed and used in this study for PCR and real-time quantitative PCR. Reference genes Pros54 and eIF3-S8 and the immune gene Dicer were selected from the *Polistes canadensis* and *P. dominula* genomes, and orthologs identified using a BLAST search on a common wasp draft transcriptome assembly. Candidate pathogen primers for KBV, Moku virus, *A. fumigatus* and *M. wisconsensis* were designed using sequences extracted from the metatranscriptomic assembly in this study. All primers were designed using PRIMER Blast

| Target | Forward | Reverse | Product Length |
| --- | --- | --- | --- |
| Reference Gene: Pros54 (RT-qPCR) | TGGTGAAATTAGCAAAACGACTCA | GAACAGTGACGAGGTGGCTA | 150 |
| Reference Gene: eIF3-S8 (RT-qPCR) | AGAAGAGTATGGCTGATGGTGA | TCACTCTCTGAATCGCTACCC | 70 |
| *Kashmir Bee Virus* (PCR) | TGATGGTGATTGTGGAGCCC | TCGACTCCCGGATAACCTGT | 690 |
| *Kashmir Bee Virus* (PCR & RT-qPCR) | ACCAGGAAGTATTCCCATGGTAAG | TGGAGCTATGGTTCCGTTCAG | 79 |
| *Moku Virus* (PCR) | ATCTGAACTGCCACCGTCAG | TGCTGCTGCCTCATTAACCA | 744 |
| *Moku Virus* (PCR & RT-qPCR) | TGGTGCGATAGCTAAGCCTG | GAACCCAGAATGGTGCTGGA | 134 |
| *Aspergillus fumigatus* (PCR & RT-qPCR) | CTAACTTTCGTTCCCTGATTAATG | CTTGGATTTGCTGAAGACTAAC | 76 |
| *Moellerella wisconsensis* (PCR & RT-qPCR) | TACCGACAGAAGAAGCACCG | CTTAATCAACCGCCTGCGTG | 112 |
| Dicer (PCR & RT-qPCR) | CAACTCCGAGATAGGTGACAGAAG | AGAAGAGGGTCCGCATCCTT | 73 |
